# Label-Free Biosensors for Laboratory-Based Diagnostics of Infections: Current Achievements and New Trends

**DOI:** 10.3390/bios10020011

**Published:** 2020-02-12

**Authors:** Boris G. Andryukov, Natalya N. Besednova, Roman V. Romashko, Tatyana S. Zaporozhets, Timofey A. Efimov

**Affiliations:** 1 Somov Research Institute of Epidemiology and Microbiology, 690087 Vladivostok, Russia; besednoff_lev@mail.ru (N.N.B.); niiem_vl@mail.ru (T.S.Z.); 2 Institute of Automation and Control Processes (IAPU) of the Far Eastern Branch of the Russian Academy of Sciences, 690041 Vladivostok, Russia; romashko@iacp.dvo.ru (R.V.R.); tim2vl@yandex.ru (T.A.E.)

**Keywords:** biosensors, label-free biosensors, laboratory-based diagnostics, infectious diseases, sensory strategies, molecular markers

## Abstract

Infections pose a serious global public health problem and are a major cause of premature mortality worldwide. One of the most challenging objectives faced by modern medicine is timely and accurate laboratory-based diagnostics of infectious diseases. Being a key factor of timely initiation and success of treatment, it may potentially provide reduction in incidence of a disease, as well as prevent outbreak and spread of dangerous epidemics. The traditional methods of laboratory-based diagnostics of infectious diseases are quite time- and labor-consuming, require expensive equipment and qualified personnel, which restricts their use in case of limited resources. Over the past six decades, diagnostic technologies based on lateral flow immunoassay (LFIA) have been and remain true alternatives to modern laboratory analyzers and have been successfully used to quickly detect molecular ligands in biosubstrates to diagnose many infectious diseases and septic conditions. These devices are considered as simplified formats of modern biosensors. Recent advances in the development of label-free biosensor technologies have made them promising diagnostic tools that combine rapid pathogen indication, simplicity, user-friendliness, operational efficiency, accuracy, and cost effectiveness, with a trend towards creation of portable platforms. These qualities exceed the generally accepted standards of microbiological and immunological diagnostics and open up a broad range of applications of these analytical systems in clinical practice immediately at the site of medical care (point-of-care concept, POC). A great variety of modern nanoarchitectonics of biosensors are based on the use of a broad range of analytical and constructive strategies and identification of various regulatory and functional molecular markers associated with infectious bacterial pathogens. Resolution of the existing biosensing issues will provide rapid development of diagnostic biotechnologies.

## 1. Introduction

Monitoring and early identification of markers is essential for the diagnosis of infectious diseases. Therefore, the study of molecular markers of biological agents, as well as the search and development of highly effective and sensitive methods for indicating pathogenic microorganisms, have always been the focus of attention of researchers [1,2,3].

As our knowledge of the complex biochemical processes underlying the pathogenesis of infectious processes deepened, it became necessary to design more sensitive and highly specific diagnostic methods. They are based on determination of molecular markers and on profiling of microorganisms without cultivation, enrichment, and isolation of pure cultures. These methods are expected to be perfect analytical tools for controlling pathogenic microorganisms and form a basis for identifying the relationship between molecular structures and biological processes [1,4,5].

The classical microbiological and immunoserological methods, as well as the modern diagnostic platforms such as enzyme immunoassay (EIA) and chemiluminescent assay, polymerase chain reaction (PCR), flow cytometry, and matrix-assisted laser desorption/ionization (MALDI) mass spectrometry, which have found practical application in recent decades, are the most widely used approaches for accurate verification of causative agents of infections at centralized laboratories of medical hospitals and centers. Currently, highly efficient and accurate techniques of molecular hybridization and amplification of nucleic acids hold a special position in clinical diagnostics of infectious diseases. However, these diagnostic tools require expensive equipment, long testing time, plenty of consumables, and skilled personnel. For this reason, they are not always affordable for small local hospitals, especially in case of limited economic resources and decentralized infrastructure of medical units [4,6,7].

The biosensor technologies that have emerged over the past decades have become innovative platforms for analytical and diagnostic research in many fields of scientific research and practical application. In particular, they have become indispensable tools for the biomedical and environmental sciences, combining high analytical characteristics, compactness, and accessibility [1,4].

Many years of world experience in using modern biosensors for specific indication of pathological biological agents have shown that these devices have a high potential to become fast and reliable in operation, highly specific and sensitive tools for timely diagnosis [3,5,6,8]. They fully comply with the modern «point-of-care testing» concept and can be successfully applied in addressing biosafety issues and emergency indication in emergency situations of natural or man-made origin to protect the population or the environment [8,9].

It is interesting that the evolution of sensory technologies developed in parallel with the emergence of diagnostic platforms for the lateral flow immunoassay (LFIA), which in our time are considered as simplified formats of modern biosensors [10,11,12,13].

## 2. Lateral Flow Immunoassay (LFIA) as Simplified Formats of Modern Biosensors

**Figure 1:**
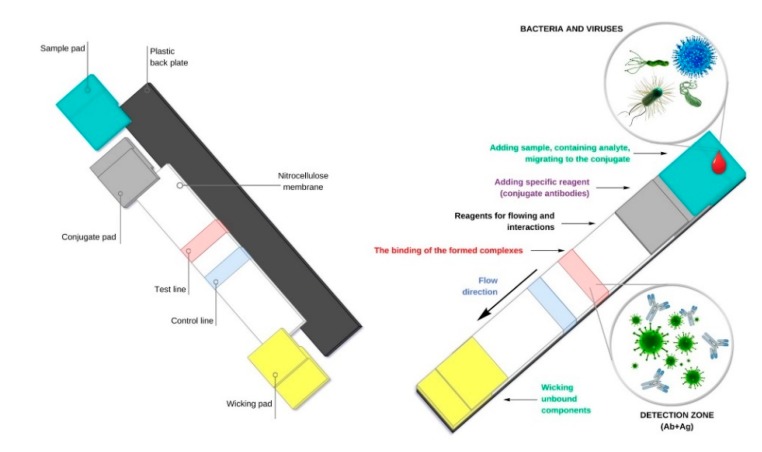
Schematic representation of the side-stream immunoassay mechanism lateral flow immunoassay (LFIA).

Among precursors of modern biosensor technologies are such diagnostic platforms as quantitative and semi-quantitative test systems for identifying specific antigens [9] and antibodies [10] as well as gene amplification products [11,12,13,14] based on latex agglutination and lateral flow immunoassay (LFIA). In the past decades, these tools have been successfully used to diagnose and identify molecular markers of infection, and they have not lost their biomedical significance today [10,11,13,15] (Figure 1).

For instance, in their recent study, C.S. Jorgensen (2015) and co-authors from Denmark successfully tried the first commercial combined test for the detection of *Streptococcus pneumoniae* and *Legionella pneumophila* antigens in urine by LFIA [14]. This increased the popularity of this versatile technology, which is equally efficient in the format of “sandwich” analysis for high-molecular-weight (HMW) antigens of microorganisms and antibodies against them in biosubstrates and for low-molecular-weight (LMW) analytes [16,17,18,19,20,21].

To date, test systems based on the LFIA technology in both standard and multiplex formats [15,17,19,22] have occupied the major part of the world’s segment of laboratory-based rapid diagnostics. Nevertheless, despite the obvious attractiveness of the LFIA technologies, the substantial disadvantages of immunoassay constrain the expansion of practical applications of these diagnostic platforms in bacterial infection diagnostics (Table 1).

The impressive achievements in the field of molecular biology, genetics, nanotechnology, as well as electronics in the second half of the 20th century became the basis for the emergence and rapid development of biosensor strategies.

Research and development of these highly selective analytical devices have become a popular and most actively developing biotechnological trend over the past 15–20 years. On the example of biosensors, the technological evolution in the field of measuring devices from semi-quantitative of the immunoassay methods to high-performance modern multifunctional analyzers is especially pronounced [21,22].

## 3. Introduction to Biosensor Technologies

The history of the biosensor technology concept began almost 60 years ago with the creation of the first device of this type for measuring blood sugar (L. Clark Jr., and C. Lyons). The authors proposed an “electrode system” based on the principle of using electrochemical detection of a product (oxygen or hydrogen peroxide) of the enzymatic glucose oxidase—glucose reaction [4].

Over the past decades, a giant path has been traveled and impressive results have been achieved both in the evolution of biosensor technologies and in expanding the scope of their application. The phenomenal advances in molecular biology, bioelectronics, nanotechnology, electronics, electrochemistry, and other sciences have formed the basis of innovative technical strategies adapted to create modern biosensors.

The emergence and use of biosensors based on integrative constructive strategies has enabled these devices in recent decades to be widely used in various fields of science, medicine, pharmacology, biological and food safety, and environmental protection. This biotechnological breakthrough was the result of the development and creation of powerful and sensitive analytical tools that use biological molecular elements as sensors [11,13,18].

Biosensors have shown great potential for use in medicine, including as a tool for the simultaneous detection in real time of several markers of bacterial and viral infections [5,6,7,23]. In addition, the possibility has arisen to conduct quantitative monitoring of the safety of the environment and the population, the detection of pathogenic biological agents in case of emergencies associated with natural, man-made accidents, or terrorist attacks [24,25].

The technical strategies used to create biosensors are diverse. The presence or absence of labels for identifying biomarkers is crucial for the division of biosensors into two structurally different types having fundamentally different properties and applications.

In this review, the authors focus on the properties and design features of the most popular label-free biosensors for biomedical research and environmental protection, optical and electrochemical devices, as well as on comparatively new microwave sensors. In addition, we will focus on modern achievements and trends in the development of these analytical platforms in the field of indication of biological agents and biosafety.

## 4. Main Types of Biosensors and Their Functions

In the modern world, the interest in biosensor technologies, fairly considered as one of the rising trends in the scientific and technical sphere, is observed to substantially increase. According to experts’ estimates, in 10–15 years the market for these analytical devices will exceed $70 billion USD [5,26,27].

According to the definition given by the International Union of Pure and Applied Chemistry (IUPAC) [28], biosensors are integrated autonomous devices that provide quantitative (semi-quantitative) analytical information about the target analyte using a biological recognition element (bioreceptor) that has a spatial relationship with the transducer.

Thus, biosensors are portable analytical devices equipped with biological elements, potentially capable of controlling biochemical parameters of physiological and pathological processes, which are inaccessible to modern analytical tools [7,29,30]. These are a kind of detectors with their action based on the specific interaction of biomolecules and bioreceptors, used for detecting and measuring minimum concentrations of various analytes [6,29,31,32].

**Figure 2:**
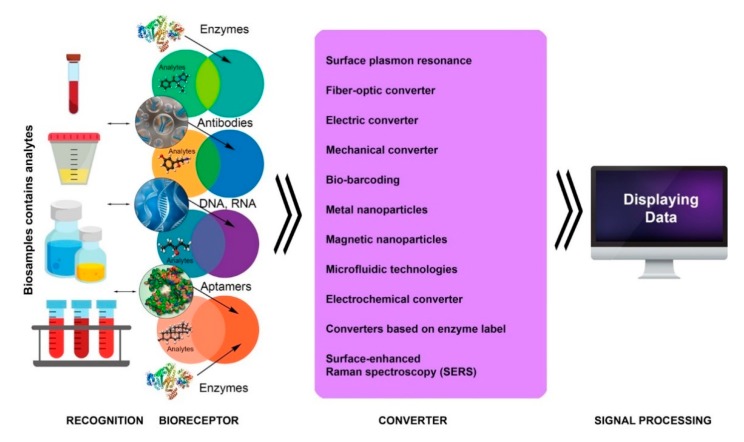
Schematic diagram of the structure of biosensors.

Structurally, biosensors are a complex consisting of three main functional elements: (I) bioreceptor (bio-recognition component) with the elements for recognizing target analytes (target molecules) contained in biosubstrates, which are located on the sensor plate; (II) transducer, acting based on physicochemical (electrochemical, spectroscopic, or optical) principles; and (III) electronic device for signal processing, recording, and displaying data in a form (analog or digital) convenient for the researcher [25,33,34,35,36] (Figure 2).

Over the decades of development of the biosensing technology, a large number of structurally different sensors and approaches to systematize them have been proposed [25,29,37,38,39,40].

To date, there exist a significant number of principles of biosensor classification depending on the nature of the biochemical component used, the analytical problems solved, the type of signal transducer, the supposed areas of application, and the generated signal.

Bioreceptors and biosubstrates, as well as reactions of interaction on a secretory panel, are a common functional element for many types of receptors. The technical approach, which is used as a basis to implement the method of detection (transduction) of signal of sensor’s interaction with biological objects, determines the basic principle to differentiate these analytical devices [25,41,42,43,44,45,46,47].

**Figure 3:**
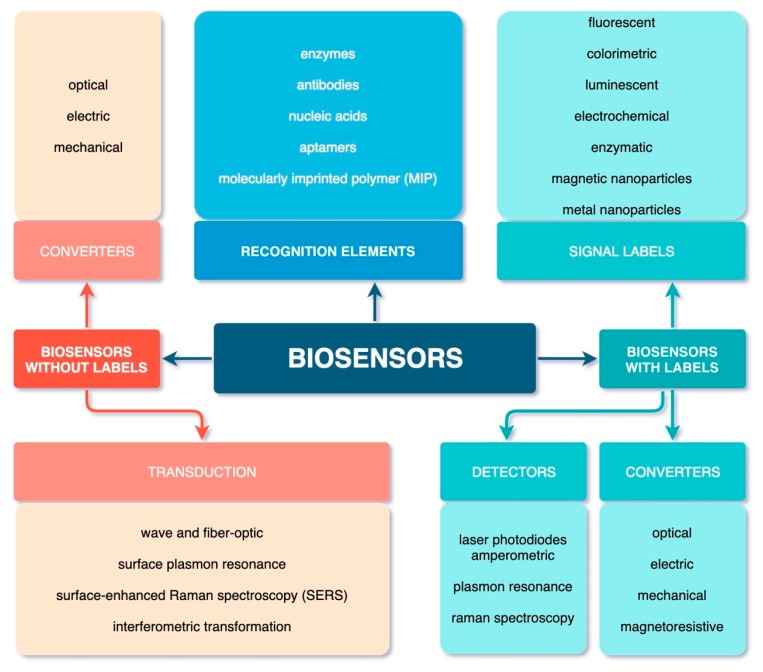
Classification of biosensors based on constructive strategies for detection methods.

According to this principle, in one of the classifications proposed by IUPAC [28], all biosensors are divided into two large groups depending on the detection method used in the technical implementation and also on the design differences used in the development of methods of detection with these analytical devices: with and without labels (markers) (Figure 3).

The following recognition elements (bioreceptors) are common to all types of biosensors used in biomedical diagnostics: immunoglobulins (antibodies), enzymes (or microbial cell homogenates), nucleic acids (DNA, RNA, PNA) [23,48,49,50], microbial cells (microorganisms) [25,51,52,53], and aptamers (short DNA and RNA oligonucleotides capable of specifically binding to certain target molecules) [3,54,55].

These receptors at normal surface concentrations (1–5 mg/mm^2^) are immobilized on a solid sensor substrate (matrix) by covalent binding or biotin–avidin interaction. They serve for selective binding and identification of target analytes (ligands) in biological fluids (whole blood, serum, plasma, urine, saliva, liquor, tissue extracts, and cell cultures) [3,5,52,53,54].

When designing highly sensitive biosensors, the correct choice of matrix and conditions for immobilization of bioreceptors is of key importance. In case of non-covalent binding, the receptor is retained on the sensory substrate of transducer by electrostatic, van der Waals, or ion interactions which hold biomolecules firmly enough. The main advantage of this type of immobilization is the lack of influence of the matrix on biological properties of the receptor [25,26]. In case of covalent binding to the surface of the sensor matrix, biomolecules are held firmly, which prevents them from being leached out of the matrix, which is of crucial importance for designing a reusable biosensor [43,45,56,57].

Nanomaterials, whose unique catalytic efficiency and adsorption properties provide optimum physical and chemical characteristics of the sensor substrate surface, are increasingly frequently used in latest-generation devices [26,43,58]. In them, neither bioreceptors nor analytes undergo conformational changes and loss of biological activity, which eventually provides an effective ligand–receptor interaction transmitted as a specific equivalent amplified signal [25,26,58,59].

The mechanism of transmission of ligand—receptor interaction signal and its transduction are other important functional elements of biosensors. The signal is transmitted using electrodes (gold, silver, platinum, mercury, etc.) with various surface modifications and graphite pastes [25,57,60,61]. This biochemical process is detected and transduced into quantitatively detectable physical parameters using one of the several types of physicochemical transducers that provide optical (reacting to changes in physico-optical parameters), piezoelectric (quartz crystal microbalance technology), electrochemical (based on the principle of electric current measurement), or micromechanical signals, which are processed at the output by a processor and analyzed [26,58,59,60] (Figure 3).

For example, in biosensors, where immobilized enzymes are used as a recognition element on the sensor plates, substrates from the biomaterial enter into a biochemical reaction with them in the presence of catalysts. The resulting product is determined with an electrode that transduces the biochemical reaction into an electrochemical signal, the magnitude of which is proportional to the amount of substrate in the biomaterial under study [22,61,62].

In recent decades, the key objectives of interdisciplinary research for designing modern biosensors (representing, in fact, the first generation of bioelectronic devices) are the improvement of the parameters of close interaction between biochemical and physical functional elements in order to increase their sensitivity and selectivity and the reduction in the limits of detection of target analytes [28,43,63]. The above-indicated characteristics of these analytical systems are of crucial importance in diagnostics of infectious diseases.

Progress in the development of biosensory diagnostics of bacterial and viral infections has been achieved mainly due to the latest improvements in the methods used for indication of specific markers [22,64,65,66,67].

In addition to the already used analytical devices, where immunoglobulins and enzymes in the form of bacterial homogenates are used as a bioreceptor, whole-cell microbial biosensors have also been put into practice in recent years. In the latter, live natural or engineered microorganisms (such as *Escherichia coli* or *Staphylococcus aureus*), integrated on a sensory substrate, assimilate target organic compounds from biosubstrates (such as, for example, antibodies from blood serum) and simultaneously act as a sensitive mechanism [25,68,69,70,71,72]. In this case, a positive reaction to the promoter of the target molecule after its transport through the cell membrane and diffusion inside the bacterial cell causes expression of the reporter gene, which is recorded as a quantitative response via optical [7,24,26,39,56] or electrochemical signals [30,48,49,50,73].

**Figure 4:**
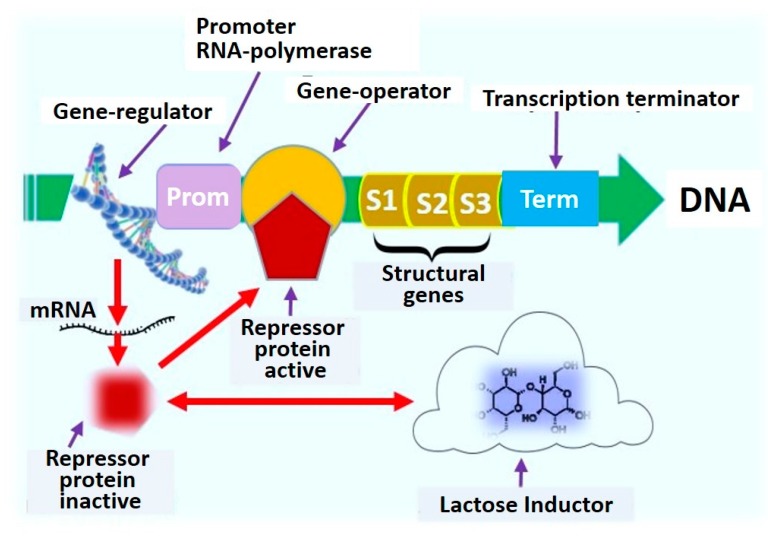
Functional diagram of the lactose lac operon of *Escherichia coli* in microbial biosensors.

The use of reporter genes to identify factors triggering genetic reactions in live microorganisms was proposed in the middle of the 20th century [37,38], when the functions of the lactose operon of *E. coli* (lac-operon) and its relationship with the patterns of metabolism and microbes’ growth were described. These fundamental findings were confirmed in subsequent years by the study of the role and structure of DNA and other reporter genes, such as xylE and tfdA, which are now actively used as a biophysical model in environmental research [23,48,49,50] (Figure 4).

Recently, X. Liu with co-authors has reported the design of a biosensor in which synthetic antimicrobial peptides were used as new recognition bioreceptors [74]. The proposed analytical device in combination with the impedancemetric method of recognition allowed rapid and quantitative indication of bacterial pathogens in biosubstrates (*E. coli*, *S. aureus*, *Pseudomonas aeruginosa*, and *Staphylococcus epidermidis*) at concentrations from 10^2^ CFU/mL. Moreover, this sensor allowed differentiation of live bacteria from dead [68,69,71,72].

Another team of researchers proposed a biosensor design for highly sensitive and rapid identification of *S. aureus*, where bacteriophage was used as a receptor with a detection range from 4 × 10^8^ CFU/mL [70].

The invention of the regulatory biosensor associated with the latest advances in molecular genetic technologies and the discovery of new mechanisms for detecting various extracellular and intracellular signals, as well as their subsequent optical and electrochemical transduction, were implemented in the corresponding types of label-free biosensors [48,49,71,72].

With the development of modern biosensor technologies, label-free biosensors are becoming more widespread for the indication of biological agents and for biomedical research.

### 4.1. Label-Free Biosensors

The widespread use of label-free biosensor was facilitated to a large extent by the fact that they use biological or chemical receptors for direct detection of the analyzed molecules in the sample, without the use of special enzyme and radioactive or fluorescent labels. They allow screening of intermolecular interactions and cellular reactions, providing detailed information about the selectivity of effect of bacterial exotoxins and the specificity of antimicrobial agents, the antigen—antibody interaction, as well as the kinetics of inflammatory process, immunological, and serological reactions [3,40,75,76].

This type of biosensors requires only one recognition element, which leads to simplification of the pattern of analysis, reducing both the analysis time and expenditures for reagents. The latest generation of label-free biosensors provides real-time quantification of products of biomolecular reaction, which makes it possible to perform continuous data recording, allowing kinetic monitoring of parameters of the ligand—receptor interaction recognition process [24,32,77,78,79,80].

Moreover, an important advantage of the use of label-free biosensors is that target analytes are detected in their natural form without labeling and chemical modification, and thus, they can be preserved for further analysis (Table 2).

In recent decades, numerous studies have been carried out to develop new types of receptors [29,63,87,88] and methods of recognition for label-free biosensors. They can generate signal immediately after binding to the recognition element and do not require additional interactions with the labels that provide signal. In this context, many physicochemical types of transducers have been proposed that transform the results of bioreceptor-based binding of selected targets (for example, increase in weight, specific resistance, or surface refractive indices), which are recognized in various ways [29,71,78,89,90].

Optical, electrochemical, electric (piezoelectric), microwave, or (micro) mechanical transducers are promising strategies for recognizing ligand-receptor interaction signals in label-free biosensors used to indicate biological agents or biomonitoring the environment [32,40,80,84]. Among the most powerful detection and analysis tools, widely used in biomedical research and in practical medicine, are biosensors with optical transducers, considered the basic tool for signal perception [3,7,75,89,90].

#### 4.1.1. Label-Free Biosensors with Optical Converter

The study of the interactions of biomolecules, the metabolic activity of prokaryotic cells, the analysis of biosubstrate samples or environmental samples for pathological biological agents are examples of important applied biomedical problems that are solved using methods that use radioactive, colorimetric, or fluorimetric labels (markers). The study of the interactions of biomolecules, the metabolic activity of prokaryotic cells, the analysis of biosubstrate samples or environmental samples for pathological biological agents are examples of important applied biomedical problems that are solved using methods that use radioactive, colorimetric, or fluorimetric labels (markers). These methods allow you to indirectly determine the analyzed substances and molecules; however, they are laborious and time-consuming. In addition, the markers used affect the passage of chemical reactions in the biodetection system and do not allow measurements in real time [7,24,39].

The use of optical label-free biosensors allows direct detection of the target molecules under study. In this case, the phenomenon of interaction of electrons with an electromagnetic field is used and this can be represented by a propagating light wave. The high sensitivity of devices of this type is mediated by the ability of biological molecules to cause a change in the speed of light transmitted through the biomaterial and register the effect of the generation of a damped wave [24,26,56,72,75].

The operation of converters in biosensors of this type is based on direct measurement of changes in optical properties in the presence of the analyte, such as absorption, reflectance, radiation, or interferometric pattern, which can be detected by a photodetector [7,26,56]. These devices have several advantages, including immunity to electromagnetic interference, the ability to perform remote sensing, high sensitivity, direct measurement in real time, and the possibility of multiplexing (simultaneous detection of several analytes). Microbial optic biosensors that detect interactions between microorganisms and target ligand targets are no exception [5,9,14,46].

The variety of detection methods and optical converters used in biosensors without labels of this type is huge; therefore, in this review, the authors limited themselves to developments that have successfully established themselves in the detection of biological agents [56,75,76].

Optical label-free biosensors can be classified according to the technologies on which they are based (for example, interference schemes, optical waveguides, photonic crystals, and others). In addition, optical ring resonators or ellipsometry can be used in them [38,56,76,82].

Advanced technologies in the design of label-free optical biosensors with an emphasis on the indication of biological agents and biomedical research are associated with the development of modern transduction methods (fiber optic and attenuated electromagnetic field systems, surface plasmon resonance, Raman spectroscopy, interferometry, using photonic crystals), as well as with new recognizing elements (molecularly imprinted polymers) [71,79,84,91,92,93].

**Figure 5:**
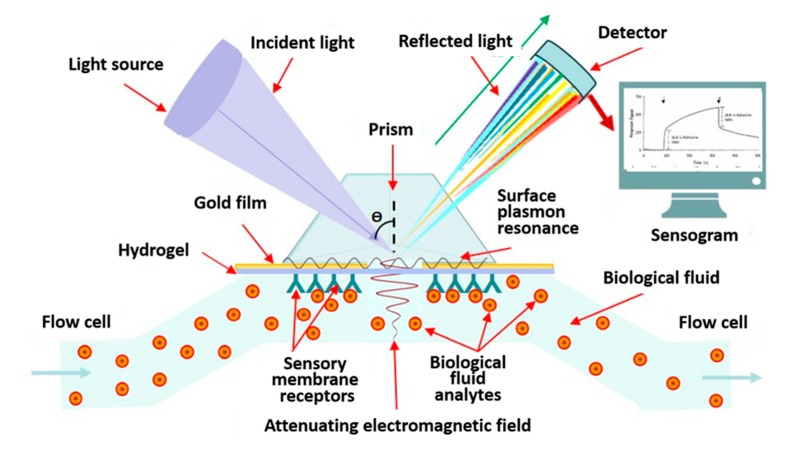
Advanced technologies implemented in the design of optical label-free biosensors.

Among the label-free biosensors using optical signal converters, it is worth highlighting devices based on surface plasmon resonance (SPR), which can have different principles for taking the output signal and have different technical characteristics (sensitivity, size, wavelength ranges, and others). Registration of SPR is based on a system containing a source of monochromatic linearly polarized light, a glass prism, a metal film on the surface of the prism, and a photodetector [78,85] (Figure 5).

A ray of light incident on the base of a prism undergoes total internal reflection for incidence angles greater than critical and is accompanied by the appearance of a damped wave propagating from the prism into the metal layer [78,83,93]. The effect of the detection of biological molecules in optical biosensors based on SPR consists in a change (increase) in the intensity of the reflected beam during the course of the reaction. SPR has become one of the common methods of biodetection, which have entered the practice of marker-free biosensor technologies [75,83,94,95].

In recent years, the main direction of research in the field of biodetection based on SPR has been the search for new methods to increase their sensitivity (for example, using Au-Ag electrode coatings, creation a periodic metal-dielectric structure on the surface of a metal film, or using long-range plasmons) [38,56,72,78,85].

#### 4.1.2. Electrochemical Label-Free Biosensors

Of particular interest are the development of electrochemical label-free biosensors among modern sensor platforms used for biomedical research.

Electrochemical label-free biosensors translate information on the chemical and biological properties of the test substances directly into an electrical signal. Electrochemical detection is based on the direct catalytic transfer of electrons between the surface of the electrode and the active center of a bio-recognizing reagent. The most popular biosensors of this type use the following measurement methods: amperometry, conductometry, cyclic voltometry, chronometry, impedance spectroscopy [48,49,50,71,82,83,84].

Catalytic electrochemical label-free biosensors have been used for a long time, but they revealed a number of disadvantages associated with the unstable activity or inhomogeneity of the enzyme, which mediates the need for calibration [48,73]. These shortcomings led to the development of a number of biomolecules with various different electrochemical properties, which led to the rapid evolution of this type of sensors and the emergence of more stable biosensors.

Most modern biosensors designed to detect infectious pathogens are also based on the use of electrochemical signal conversion. They are designed by modifying the surface of metal and carbon electrodes using various recognition elements (for example, enzymes, antibodies, DNA, aptamers). These sensors are based on measuring the output signal that is usually generated as a result of specific binding reactions or catalytic reactions on the electrode surface [49,71,79,82].

The need to develop electrochemical sensors has become especially urgent for biological agent indication systems in which early detection or monitoring (for example, environmental safety, biosafety, or monitoring molecular markers of the infection process) is of great importance [71,73,96,97,98].

In modern electrochemical label-free biosensors for the detection of bacterial pathogens, much attention is paid to the construction of bioelectrodes using various materials. Among the most widely used materials are thin polymer films, nanostructured metal oxides, self-organizing monolayers of organic molecules (SAM), and carbon nanostructures (nanotubes, fullerenes, graphenes) [50]. For example, SAMs are considered ideal material for immobilizing nucleic acids when constructing biosensors for detecting bacteria [50,84,98,99].

The prospect of electrochemical label-free biosensors is undeniable, since they do not require complex or expensive equipment, can be used in the field, and even be implanted into the human body for continuous monitoring of various biologically active compounds [48,71,73,96].

In addition, biosensors of this type have significant cost-effectiveness, high sensitivity, and a large range of linearity of detection. They are able to work with small volumes of samples, and the result does not depend on the turbidity of the studied samples, in contrast to optical methods based on spectroscopic, impedancemetry, and bio-layer interferometry transduction [48,49,50,100,101].

To expand the analytical capabilities of electrochemical biosensors, work is underway to improve the methods of immobilization of the biocomponent on the electrode, to miniaturize the sensor elements, and to increase their stability. The main limitation of the use of biosensors in medicine, biomedical research, and environmental protection is associated with the need to use one type of sensor to determine only one compound [50,71,73,96].

The recognition elements used in these types of analytical devices include DNA, RNA, PNA, and aptamers [3,19,31,48,49,50,55]. For example, the sensor technology of DNA hybridization, which, relying on electrochemical (impedance spectroscopy) and optical methods, recognizes the complementary target DNA chain of a pathogenic microorganism, is becoming increasingly popular today [49,50,54].

Due to the ease of use and high sensitivity, electrochemical and optical label-free biosensors have been most commonly deployed for specific and non-specific indication of biological agents in recent years (Table 3).

#### 4.1.3. Microwave Label-Free Biosensors

In the category of label-free biosensors, microwave biosensors (MW-biosensors) occupy a special place due to their unique properties, which significantly distinguish them from both optical and electrochemical sensors.

The methodology of constructing microwave label-free biosensors (MW-biosensors, MW-sensors) is based on the dielectric property of biological cells (electrical conductivity when an electric field is applied). These properties play an important role in cell life and are mediated by the presence of water, macromolecules dissolved in it, as well as the localization of intracellular structures of prokaryotes and eukaryotic organisms. Moreover, the dielectric properties of cells underlie intercellular interaction in complex multicellular biological systems or in bacterial populations, and therefore are unique and specific to every living organism [69,107,108].

Therefore, the permittivity of bacterial cells on the influence of external electric fields depends on their species, type (gram-positive or gram-negative) [107,109,110], as well as on the electrical conductivity and permittivity of various intracellular components of bacteria. In addition, they may depend on the physiological state of bacteria (metabolic activity level, degree of viability), as well as on living conditions and the state of their hydration [111,112,113].

For example, M. Biagi with colleagues found that, depending on the state of a biological sample (wet biosubstrates, water samples, or dry environmental objects), *E. coli* bacterial cells have different permittivity values: from 4 GHz (dry samples) to 20 GHz (wet samples) [107].

Based on the dielectric properties of the specific signatures of the biomolecules that make up the bacterial cells, label-free MW-biosensors were designed for the specific indication of biological agents for medical diagnostics, biomedical research, environmental protection and biosafety [111,114]. The biological target of these devices can be specific biomarkers of the infectious process, as well as macromolecules (proteins, nucleic acids). Monoclonal antibodies, DNA, aptamers, and bacteriophages are usually used as recognition elements (bioreceptors) immobilized on the sensor surface [114]. The detection and indication of biological agents in MW-sensors is based on a change in the dielectric constant of the detection surface during the interaction of target biomarkers with immobilized bioreceptors.

In the category of label-free MW-biosensors, sensors occupy a significant place due to their unique properties. Unlike optical biosensors, which measure the optical properties of biomolecules (for example, by interferometry, PPR or SERS), MW-biosensors use capacitive sensing (low-frequency or microwave) to measure permittivity using counter capacitors, resonators, and microstrip structures [114,115]. The high sensitivity and specificity of MW-biosensors allow not only to identify biological agents but also to assess the physiological state of bacterial cells, to differentiate living cells from dead cells [69,113,114].

Unlike electromagnetic biosensors, MW-sensors are attractive for their minimally invasiveness and cost-effectiveness [69,115].

In addition, MW-biosensors differ from optical and electrochemical label-free sensors in that they provide a wide format of information and expand the possibility of specific and non-specific indication of biological agents. Given the high sensitivity and specificity, this quality makes these devices promising for the medical diagnosis of infectious diseases, environmental monitoring, and biosafety control.

Convincing evidence of the high detection rate and impressive sensitivity of MW-biosensors was given by S. Oberoi with colleagues. A biosensor of this type constructed by the authors detected and identified 4–5 min before 1–2 colony forming units (CFU) of *E. coli* in water samples [113].

Of undoubted value and high relevance is the work on the study of the possibility of label-free microwave sensors. The authors tested a MW-sensor that detected different concentrations of *E. coli*, reaching 10^3^ CFU/mL with biofunctionalization of the sensor by polyclonal antibodies [69,113].

Unlike other sensors, MW-biosensors are relatively new biosensing tools. However, they have already successfully shown themselves in the diagnosis of cancer, endocrine, and heart diseases and increasing the effectiveness of microbiological practice. In a recent study by R. Narang with colleagues, the possibilities of a microfluidic MW-biosensor to control the growth dynamics of *E. coli* were investigated. The sensor device demonstrated an almost instantaneous response to the dynamics of changes in bacterial concentrations in various pH solutions by screening for changes in the resonance amplitude and frequency characteristics of the microwave system [114]. Thus, the use of this sensor opens the possibility of a quick study of sensitivity to antibiotics, which usually takes 1–2 days.

Thus, the detection speed, non-contact, non-invasiveness, high sensitivity, and efficiency of MW-biosensors give them a wide prospect of application for continuous monitoring (for example, environmental biomonitoring) and solving biomedical tasks.

### 4.2. Mechanical Biosensors

Biosensors that use the mechanical resonance phenomenon for the detection of biological objects represent a separate class. Such biosensors include, primarily, quartz crystal resonators, micro- and nano-electromechanical systems [115,116].

The first attempts to create piezoquartz biosensors operating immediately in liquid media were unsuccessful, since quartz crystal stopped oscillating when immersed in a solution. A technique that made it possible to measure concentrations of biological objects in liquids was as follows: The sensor was kept in the analyzed solution, then dried, and the measurement of the quantity of attached biological objects was performed already in the gas medium [117]. This method was called “dip and dry” [118]. Subsequently, an approach was found that provided real-time measurements also in a liquid medium [119].

A fundamentally new pattern of excitation and detection was the use of a flow-through cell which ensures contact of the analyzed solution with only one side of the quartz crystal [119]. The advantage of this method is the opportunity of its application in the development of immunosensors as rapid and simple test tools. By extending the time of sensor’s exposure to the analyzed sample, thorough washing and drying, the reliability of the determination results can be increased, and the detection limit decreased to extremely low, 100 fmol in this case. Despite the relatively low reproducibility of the results, this method has not lost its relevance to date.

Currently, biosensors based on micromechanical resonators, referred to as cantilevers, are actively developed. Adsorption of biological objects on the surface of a microcantilever can lead to its bending due to the effect of surface tension forces or a shift of its resonant frequency caused by weight variations [117]. Depending on whether the magnitude of the static bending of a cantilever or the frequency of its oscillations is detected, the mode of biosensor operation is divided into static and dynamic. When operated in the static mode, the cantilever bends as a result of variations in the surface tension forces on one of the cantilever’s surfaces caused by any molecular reaction.

To date, there is no satisfactory theoretical model to precisely describe the factors and phenomena responsible for changes in the occurrence of forces that cause a cantilever to bend. The lack of a theoretical model is explained by a rather complex nature of equilibrium between the sensitive layer and the surrounding water molecules, voluminous solution, target molecules, ions, etc. [120,121].

Most frequently, deflection of a cantilever is measured using a laser beam reflected from it. In addition, electrical (piezoresistive) methods are also used to measure binding of proteins and DNA molecules. Single cantilevers are sensitive to parasitical factors affecting the magnitude of bending. Such false positives can occur due to the proximal variations in the biosensor, refractive index of the liquid or its layers, temperature, or inhomogeneities in the liquid. Number of false positives can be reduced through differential measurements, which allow on-site determination of the difference between the induced tension on the functionalized cantilever and the cantilever that has been put into the passive state. The sensitivity of such sensors ranges from hundreds of picomoles [122] to several nanomoles [123].

A biosensor designed using cantilever-bending sensors, piezoquartz resonator, WGM resonator, and the surface plasmon resonance method implies the presence of some sensory region whose minimum size is limited to usually dozens or hundreds of microns. Reducing the size of the sensor causes its sensitivity to decrease. In turn, the sensitivity of a cantilever-based biosensor operating in the dynamic mode increases with the decreasing sensor size. In the dynamic mode, the frequency of cantilever’s own oscillation is inversely proportional to the variation in weight of objects adsorbed on its surface. This method of measuring mass is called resonance microweighting [124].

At present, sensors based on resonant microweighting are capable of measuring the change in mass attached to objects cantilever with an accuracy of several attograms [125]. Moreover, the use of the resonant microbalance method can significantly reduce measurement time. Thus, in the study of [126] on biofilms of *E. coli* colonies, it was demonstrated that the time of detection of variations in the weight of microcultures grown immediately on the cantilever, due to their matter exchange with the environment, was reduced from 1 day to 1 h.

It should be noted that the highly sensitive weight detection based on the shift of resonant frequency requires a high Q-factor of the mechanical oscillator, which, in turn, is greatly reduced for a mechanical sensor operating in a liquid due to the viscosity in the latter, which entails a significant attenuation of resonator’s oscillations. However, operation at a high frequency increases the effect of the Reynolds number (Reynolds number, is a quantitative characteristic of the ratio of nonlinear and dissipative terms in the equation describing the propagation of a wave of finite amplitude) and allows the mechanical biosensor to operate in a liquid with a high Q-factor [125].

Nevertheless, reducing the cantilever size to micrometers and submicrometers makes it increasingly difficult to detect its oscillations, since the probing light beam undergoes strong scattering and diffraction on a small object. Therefore, such biosensors are currently designed on the basis of Fabry-Pérot resonators, which are not as demanding of size of the biosensor’s reflecting surface and show by orders of magnitude greater sensitivity to sensor’s movements.

An original alternative to immersing a mechanical oscillator in a liquid is to hold the liquid in channels made immediately on the oscillator. Such suspended microchannel resonators (SMRs) can perform measurements in vacuum, where the Q-factor may reach 15,000. Measurements of the dissipated energy of SMR devices in a liquid suggest some degradation of Q-factor that may take place in liquid-filled nanochannels [127].

Despite the high Q-factor, the efficiency of SMR biosensors still remains low mainly because of the impossibility to reduce the cross-section of liquid channels, which limits the potential of decreasing the size of SMR biosensor and increasing its sensitivity. As noted above, the detection system of cantilever movements plays a crucial role in the design of micromechanical cantilever-based biosensors. Optical, piezoresistive, and piezoelectric systems are the most common ones. Piezoelectric and piezoresistive methods consist in sputtering a thin layer of piezoelectric material on a cantilever [128]. The applied layer increases stiffness, thus, raising the Q-factor of the oscillator, which has a positive effect on sensitivity of the biosensor. In sensors with the applied piezoelectric layer, the sensitivity to variations in the attached mass reaches 175 Hz/pg [96].

A disadvantage of the piezoresistive and piezoelectric methods of detection of cantilever movements is their susceptibility to external electromagnetic noise due to the electrical nature of the physical mechanism of detection. Moreover, the sensors themselves cause excitation of electrostatic forces in the analyte under study, and this can induce false triggering of the sensor, which is not typical for optical methods of detection of micromechanical movements.

Most of modern biosensors designed to detect infectious pathogens are based on the electrochemical signal transduction. These sensors measure variations in current, electrochemical potential, and impedancemetry as a way of transduction of biochemical reactions [50,68].

In modern electrochemical biosensors for detecting bacterial pathogens, much attention is paid to the design of bioelectrodes with various materials applied. Among the most widely used materials are thin polymer films, nanostructured metal oxides, self-assembled monolayers (SAM) of organic molecules, and carbon nanostructures (nanotubes, fullerenes, graphenes) [55]. For example, SAM is considered a perfect material for immobilization of nucleic acids in designing biosensors to detect bacteria [55,72,98].

The advantages of these devices include their potential for a portable design and the simple measuring equipment. In addition to their cost-effectiveness, high sensitivity, and broad range of linear detection, electrochemical sensors can operate with a small volume of sample; the obtained result is not affected by turbidity of samples, unlike optical methods based on spectroscopic transduction [47,68].

## 5. Conclusions

The increasing number of studies in recent years associated with label-free biosensors indicates the continuous development of these technologies and detection methods. The combination of molecular biology with nanotechnology opens wide prospects for the creation of new biosensor platforms with highly effective, sensitive, and selective detection of pathogenic biological agents.

Modern trends in the development of label-free biosensor technologies are associated with the creation of new materials for the construction of transducers and the creation of conditions for a more efficient ligand-receptor interaction. Among the most attractive and promising molecular recognition elements in recent years, nanobodies (single-domain antibodies, sdAb), which have a high potential for biomedical research and applied diagnostic use, have been actively used. Like classical antibodies, nanobodies are capable of efficiently and selectively binding to a wide range of specific antigens. The uniqueness of these recombinant antibodies is due to the absence of a light chain in the structure and shortened by one domain (CP1) by a heavy chain, small size (about 15 kDa) and a number of other structural features that provide less nonspecific sorption and the ability to recognize conformational epitopes hidden for classical antibodies [129,130].

The relative simplicity of the production of nanobodies in heterologous systems (bacteria, yeast), high stability, and selectivity of binding to target ligands made nanobodies promising for the role of recognition elements in biosensor systems. In addition, the small size of single domain antibodies is a significant advantage when creating diagnostic platforms with a high degree of sensitivity with a minimum sample volume [129,131,132]. These qualities make sdAb not only an alternative to classical antibodies but for the most part they allow them to achieve analytical or diagnostic characteristics that are unattainable with conventional antibodies [130,133].

This review focuses on a large group of label-free biosensors, which are more affordable and do not require a tag for the reproduced signal but have fairly complex transduction systems. Nevertheless, the future lies in simpler and more portable diagnostic analytical systems that do not require complex conversion platforms (SPR or SERS), capable of detecting several pathogens based on multiplex formatting at once. Such biosensors can solve the global task of detecting biological agents, protecting the environment, and preventing biological threats.

Modern interdisciplinary solutions based on the innovative achievements of biotechnology, bioengineering, electrical engineering, and electronics have made it possible to develop label-free biosensors for applications in biomedical research and environmental science.

## Figures and Tables

**Table 1:** Advantages and disadvantages of test-systems based on the lateral flow immunoassay platform.

Advantages	Disadvantages	References
◾ Cheap, rapid, inexpensive, and easy to apply tests. ◾ Long shelf-life of test systems. ◾ Test systems do not require special temperature conditions for storage. ◾ No additional special equipment is required. ◾ They do not need qualified personnel. ◾ They can be used by general practice physicians or patients at home. ◾ Visual result is clear and easily distinguishable. ◾ Tests are usually sold as kits with a set of all the items needed to perform the test. ◾ Possible increase in sensitivity of test systems by the use of plasmon resonance, surface-enhanced Raman scattering (SERS), chemiluminescent or fluorescent labels. ◾ Possibility of multiplexed formats of test systems	◾ Suitable only for primary screening and require confirmation of positive results by independent methods. ◾ Special equipment (scanners, reflectometers, CCD cameras) and software are required to obtain quantitative results. ◾ Technological improvement of the method increases cost and duration of the analysis. ◾ In a competitive format, response negatively correlates with concentration. ◾ Possible technical errors in application of specimen may affect the accuracy and reproducibility of result. ◾ Increase in sensitivity of tests is based on the use of gold, silver, or enzyme nanoparticles, which limits shelf-life, increases cost of analysis, and breaks the one-step rule of application. ◾ Tested specimen must be in the form of a solution. Preliminary dissolution of dry specimens is mandatory. ◾ When the analyte content in the solution is low, the specimen needs to be concentrated.	[14] [17,21] [15,19] [16,18,20] [18,19,20] [16,18,20] [13] [14,16,17] [18] [13,15,16] [18,19]

**Table 2:** Advantages of modern label-free biosensors over similar label-based analytical techniques.

Advantages	References
● A simplified pattern of analysis.	[3,29,49,51,81]
● Reduced analysis time (rapid response time).	[7,29,82]
● Lower cost of analysis.	[7,28,80]
● Reduced consumption of organic solvents.	[33,64,78,83]
● Portability and small dimensions.	[33,43,73]
● No need in qualified medical personnel.	[3,7,39,64,83]
● Opportunity to quantify biomolecules in real-time mode.	[25,26,78,84,85]
● Target analytes are detected in natural forms, without. modifications and labels.	[22,33,73,80,82]
● High sensitivity.	[22,25,26,43,64,85,86]
● Direct measurement of analytes.	[43,51,64,80]
● Opportunity to detect small molecules.	[3,7,25,26,43,79]
● Opportunity of multiplexing.	[28,29,64,83]
● Access to kinetic and thermodynamic parameters.	[22,26,39,80,86]

**Table 3:** Examples of modern designs of label-free biosensors for specific and non-specific indication of biological agents.

Recognizing Bioreceptor	Conversion Method	Test Models of Pathogens, Sensitivity	References
Bacteriophage	Photoluminescence	*S. aureus* 4 × 10^8^ ufc/mL	[70]
Antimicrobial peptides	Impedancemetry	*E. coli*, *S. aureus*, *P. aeruginosa*, *S. epidermidis*, 10^2^ ufc/mL	[75]
Antibacterial nanoparticles Zn-CuO and graphene oxide Man/MUA-MH/Au *	Impedancemetry and electrochemical impedance spectroscopy	*E. coli*, *S. aureus* 50 ufc/mL and antibacterial effect 100% (30 min)	[93]
Thiolated protein G on: - gold electrodes - gold nanoparticles	Cyclic voltammetry and electrochemical impedance spectroscopy	*S. typhimurium*, 2.16 × 10^6^ ufc/mL *E. coli*, 50–10^3^ ufc/mL	[102]
Enzymes	Electrochemical	*E. coli O157:H7* 150 ufc/mL	[68]
Nucleic acids (DNA, RNA)	Electrochemical	*S. aureus*, 140 ufc/mL *S. typhimurium*, 48 ufc/mL	[72]
Nucleic acids (DNA, RNA)	Electrochemical	*S. aureus*, *M. tuberculosis*	[73]
Aptamer on AuNP	Autofluorescence quenching	*S. typhimurium*, 48 ufc/mL	[92]
Monoclonal antibodies	Optical	*S. enteritidis*, 80 ufc/mL *Listeria monocytogenes*	[103]
Thiolated aptamer	Impedancemetry	*Shigella dysenteriae*	[104]
Nucleic acids (DNA, RNA)	Electrochemical impedance spectroscopy	*M. tuberculosis*	[50]
Monoclonal antibodies	Surface plasmon resonance	*Enterococcus faecalis*, 10^4^–10^8^ ufc/mL	[99]
Aptamer	Impedancemetry	*Bacillus cereus*, 10^4^–10^6^ ufc/mL *Bacillus anthracis* (spores)	[32]
Nucleic acids (DNA)	Cyclic voltammetry and electrochemical impedance spectroscopy	*Salmonella* spp.	[81]
Enzyme Simulator (Graphene Quantum Dots, GQD)	Electrochemical	*Yersinia enterocolitica*, 5 (milk)–30 (serum) ufc/mL	[105]
Monoclonal antibodies	Surface plasmon resonance	*S. aureus*, 224 ufc/mL, 30 min	[71]
Monoclonal antibodies	Visualization	*Salmonella enteritidis*, 10^2^–10^8^ ufc/mL	[88]
DNA, aptamer	Electrochemical	Bird flu virus H5N1 (AIV)	[48]
Nucleic acids (DNA)	Electrochemical impedance	Zika virus, 25.0 ± 1.7 hМ.	[49]
Aptamer (rGO-TiO_2_)	Electrochemical	*S. enterica* Typhimurium, 10^1^–10^8^ ufc/mL	[98]
Nucleic acids (DNA)	Piezoelectric	*Clostridium difficile*, sensitivity 95% and specificity 95%	[90]
Monoclonal antibodies	Surface plasmon resonance	*M. tuberculosis*, 10^2^–10^6^ ufc/mL	[93,101]
Aptamer	Fluorescent	*S. enterica* Typhimurium, 6–10 ufc/mL	[92]
Monoclonal antibodies	Potentiometry	*S. enterica* Typhimurium, 6 ufc/mL	[91]
Nucleic acids (DNA)	Electrochemical impedance	*M. tuberculosis*, 10^2^–10^6^ ufc/mL	[50]
Aptamer (RNA)	Fluorescent	*S. aureus*, 10^2^–10^6^ ufc/mL	[3]
Nicolson-Ross-Weir method	Dielectric spectroscopy	*Bacillus Subtilis,* 2.10–1.30 × 10^9^ ufc/mL *E. coli* 1.60–1.00 × 10^9^ ufc/mL	[106]

Note: * Man/MUA-MH/Au—mannose/11- Mercaptoundecanoic acid/6-mercapto-hexanol/gold.
